# Subchronic Toxicity of 2,4‐Dichlorophenoxyacetic Acid (2,4‐D) to Early Life Stages of Fish

**DOI:** 10.1002/etc.4425

**Published:** 2019-06-28

**Authors:** Jeffrey Giddings, Cliff Habig

**Affiliations:** ^1^ Compliance Services International Lakewood WA USA

To the Editor:

Several products containing 2,4‐dichlorophenoxyacetic acid (2,4‐D), 2,4‐D amine salts, and 2,4‐D butoxyethanol ester are registered by the US Environmental Protection Agency (USEPA; 2005) for control of aquatic weeds, particularly invasive species such as Eurasian water milfoil (*Myriophyllum spicatum*) and water hyacinth (*Eichhornia crassipes*). The US Army Corps of Engineers (2012) has identified 2,4‐D as an efficient, cost‐effective, and selective tool for control of invasive weeds and improvement of natural habitat for fish and other aquatic organisms. However, a recent publication in this journal (Dehnert et al., 2018) suggests that current permitting regulations, which are based on testing following standard guidelines and conducted under good laboratory practice, should be re‐evaluated based on new, conflicting data obtained using nonstandard (and non–good laboratory practice‐compliant) testing. These authors present the results of fish early life stage testing on fathead minnow (*Pimephales promelas*) with two 2,4‐D amine formulations and technical grade 2,4‐D acid, which they interpret as indicating possible impacts on recently hatched fish. However, we have concerns about several aspects of the study conduct and interpretation, as discussed below, and suggest that the data do not support the authors’ conclusion about potential risks to fish of 2,4‐D use for aquatic weed control.

The 2,4‐D product labels provide instructions for 3 general types of aquatic uses: applications to weeds on banks of canals and ditches, applications to control emergent aquatic weeds, and applications to control submerged weeds. According to the product labels, target water concentrations are 2 ppm (mg/L) of 2,4‐D acid equivalents (a.e.) for typical conditions and 4 mg a.e./L for difficult conditions. (The Dehnert [] et al., 2018 study does not mention a.e., and it is not clear whether the concentrations reported reflect 2,4‐D a.e. or 2,4‐D amine.) Generally, aquatic weed control with 2,4‐D involves a single application/season, although a second application, at a minimum interval of 21 d, is permitted for some uses. A key aspect of aquatic herbicide use, including 2,4‐D, is to minimize possible oxygen depletion effects on fish from decaying treated weeds; therefore, labels recommend treating only portions of a water body at a given time and treating other areas at a later date.

Dehnert et al. (2018) evaluated effects of 2 commercial amine formulations, Weedestroy AM‐40 (WAM40) and DMA 4 IVM (DMA4), as well as technical grade 2,4‐D acid, on hatching, survival, and growth (length and weight) of fathead minnows for 30 d post hatch in a total of 3 experiments. One experiment involved exposing parental fish plus eggs and larvae, and the other 2 experiments involved exposing fertilized eggs (embryos) and larvae. For each exposure scenario, the test design included a control group and 3 test concentrations (0.05, 0.5, and 2.0 mg/L); a standard fish early life stage study consists of 5 geometrically spaced test concentrations plus a control group (US Environmental Protection Agency, 2016b). Dehnert et al. (2018) used a flow‐through exposure system consisting of 12 10‐L tanks/test or control group. The flow rate (250 ml/min) used was unusually high—the authors state that it was 24 volume replacements/d, but based on the reported flow rate and tank volume, it was actually 36 volume replacements/d, or approximately 7‐fold higher than USEPA recommendations for fish early life stage testing (US Environmental Protection Agency, 2016b). It is not clear whether the abnormally high flow rates impacted the test results, but this is possible. Test concentrations were not chemically monitored using standard chromatographic techniques; instead, test concentrations in some aquaria (~25% of the tanks for each concentration) were periodically measured using an immunoassay method (enzyme‐linked immunoassay [ELISA]). The authors did not indicate whether quality control samples were included in the analyses to evaluate the adequacy of the analytical methodology, nor whether any of the ELISA results were confirmed using standard gas chromatography or liquid chromatography analytical methodology. The reported results indicated relatively high variability in measured test concentrations among replicates and over time for a number of the test concentrations. Moreover, the reported mean measured concentrations ranged from 58% to 129% of nominal; the generally acceptable range for mean measured test concentrations in aquatic tests (indicating proper function of the exposure system) is 80–120% of nominal, with exceptions contingent on similar differences (e.g., concentrations below 80% of nominal) at all test concentrations (US Environmental Protection Agency, 2016a).

The most disconcerting aspect of the Dehnert et al. (2018) data is the nonmonotonic or inverse concentration–response relationships that occurred in many of the exposures to WAM40, DMA4, and 2,4‐D acid. In particular, these inconsistent response data were observed for larval survival, which was the only measured endpoint with statistically significant negative differences between 2,4‐D treatments and controls. In Experiment 1 (exposure of adults, eggs, and larvae), larval survival was significantly lower than that of controls at all WAM40 concentrations, and the authors identified the no‐observed‐effect concentration (NOEC) as less than 0.05 mg/L; however, mortality was negatively correlated with exposure concentration. (Similar results were reported by DeQuattro & Karasov [2016], for DMA4, with significantly lower survival at 0.05 mg/L than controls but no significant differences at 0.5 or 2 mg/L.) In experiments 2 and 3 (exposure of embryos and larvae), survival was significantly lower than controls at 0.5 mg/L WAM40, DMA4, and 2,4‐D acid, but was greater at 2 mg/L than at 0.5 mg/L in all cases. Thus, although apparent reductions in larval survival were reported at 0.05 or 0.5 mg/L in several experiments, survival was greater—often not significantly below controls—at 2 mg/L in every case. These trends suggest that the observed statistical differences in larval survival were not definitively caused by 2,4‐D exposure.

Inconsistent concentration–response trends for growth (length and weight) raised other questions concerning potential confounding factors affecting the results. Some of the observed results indicated no difference from the controls at the lower 2 concentrations, but significantly higher growth at the highest concentration (2.0 mg/L). As an attempt to explain the study results, Dehnert et al. (2018) included a discussion on possible endocrine disruption effects. This discussion is purely speculative, because the study did not evaluate any endocrine‐specific endpoints. However, 2,4‐D has undergone the USEPA's standard fish short‐term endocrine disruption testing with fathead minnows, with the only significant effect being a reduction in adult female fecundity at the highest test concentration (96.5 mg a.e./L); there were no effects on the multiple endocrine‐specific endpoints evaluated in that study (Marino et al., 2010). Until an adequate explanation can be provided for the Dehnert et al. (2018) results, it would be irresponsible to use them as the basis for regulatory decisions concerning the use of 2,4‐D for aquatic weed control.

Dehnert et al. (2018) suggest that their study was the first of its type (exposure of eggs, hatchlings, and juvenile fish) using 2,4‐D. However, fish early life stage testing following USEPA guidelines (US Environmental Protection Agency, 2016b) was conducted more than 25 yr ago using technical 2,4‐D acid, 2,4‐D amines, and 2,4‐D esters. The NOECs for fish early life stage testing with 2,4‐D acid and 2,4‐D amines range from 14.2 to 63.4 mg a.e./L (Mayes et al., 1990; US Environmental Protection Agency, 2005); the Dehnert et al. (2018) results, with a NOEC of less than 0.05 mg/L for WAM40 based on larval survival, are inconsistent with these guideline‐compliant studies.

Finally, Dehnert et al. (2018) speculate that fish could be exposed to toxicologically significant 2,4‐D concentrations in the water column for an extended period of time following application. However, 2,4‐D undergoes rapid degradation under aerobic aquatic testing (half‐life of 15 d) and is also susceptible to aqueous photolysis (half‐life of 12.9 d; US Environmental Protection Agency, 2005). These data indicate that 2,4‐D degrades rapidly in aquatic systems and is not persistent.

Overall, the study by Dehnert et al. (2018) does not satisfy the USEPA's guideline requirements for fish early life stage testing for multiple reasons, including an inadequate number of test concentrations, an excessive flow rate, water hardness outside of the acceptable range, continual aeration of the exposure tanks, and unreported analytical results for quality control samples. Given the significant deviations from USEPA guideline testing criteria, as well as the inconsistent concentration–response patterns, the study should not be relied on for regulatory decision‐making on the use of 2,4‐D to control aquatic weeds.

## Disclaimer

The views and opinions expressed in this letter are those of the authors.
